# Glycosaminoglycans modulate C6 glioma cell adhesion to extracellular matrix components and alter cell proliferation and cell migration

**DOI:** 10.1186/1471-2121-6-31

**Published:** 2005-08-19

**Authors:** Claudia Beatriz Nedel Mendes de Aguiar, Bruno Lobão-Soares, Marcio Alvarez-Silva, Andréa Gonçalves Trentin

**Affiliations:** 1 Departamento de Biologia Celular, Embriologia e Genética, Centro de Ciências Biológicas, Universidade Federal de Santa Catarina, Florianópolis, Brazil; 2 Faculdade de Medicina de Ribeirão Preto, Hospital das Clinicas de Ribeirão Preto, Universidade de São Paulo, Ribeirão Preto, Brazil

## Abstract

**Background:**

Adhesion to extracellular matrix (ECM) components has been implicated in the proliferative and invasive properties of tumor cells. We investigated the ability of C6 glioma cells to attach to ECM components *in vitro *and described the regulatory role of glycosaminoglycans (GAGs) on their adhesion to the substrate, proliferation and migration.

**Results:**

ECM proteins (type IV collagen, laminin and fibronectin) stimulate rat C6 glioma cell line adhesion *in vitro*, in a dose-dependent manner. The higher adhesion values were achieved with type IV collagen. Exogenous heparin or chondroitin sulfate impaired, in a dose-dependent manner the attachment of C6 glioma cell line to laminin and fibronectin, but not to type IV collagen. Dextran sulfate did not affect C6 adhesion to any ECM protein analyzed, indicating a specific role of GAGs in mediating glioma adhesion to laminin and fibronectin. GAGs and dextran sulfate did not induce C6 glioma detachment from any tested substrate suggesting specific effect in the initial step of cell adhesion. Furthermore, heparin and chondroitin sulfate impaired C6 cells proliferation on fibronectin, but not on type IV collagen or laminin. In contrast, both GAGs stimulate the glioma migration on laminin without effect on type IV collagen or fibronectin.

**Conclusion:**

The results suggest that GAGs and proteoglycans regulate glioma cell adhesion to ECM proteins in specific manner leading to cell proliferation or cell migration, according to the ECM composition, thus modulating tumor cell properties.

## Background

Glioblastoma multiforme (GBM) is the most common malignant form of glioma, and resistant to therapeutic interventions, causing most patients to die within 1 year after diagnosis[1]. The rat C6 glioma cell line, originally produced by Wistar-Furth rats exposed to N,N'-nitroso-methylurea, is morphologically similar to GBM when injected into brain of rats and has been used as both *in vivo *and *in vitro *model for the study of this kind of tumor[2].

Extracellular matrix (ECM) components control many aspects of cell behavior, such as differentiation, proliferation, cell morphology and attachment[3]. It has been suggested that adhesion to ECM *in vivo *and *in vitro *is involved in the earlier steps of tumor progression, resulting in genomic instability which allows for the accumulation of multiple mutations[4]. Transformation of cells is frequently accompanied by a change in adhesive properties, often resulting in a decreased adhesion of these cells[4]. Moreover, glioma proliferation and migration can be modulated by adhesion to different substrates [5-8] and they have been suggested as mutually exclusive behavior in astrocytoma cell lines[9]. In particular, C6 glioma cells have been shown to secrete laminin-2[10], and upon nerve growth factor stimulation, tenascin and fibronectin[11]. Integrins, the receptors to ECM proteins, participate in and regulate cell-substrate adhesion[4]. Adhesion to ECM proteins could direct integrin signaling with subsequent activation of cytoplasmic tyrosine kinases and induction of downstream effector pathways[12]. It has been shown that C6 glioma cells express the well-characterized fibronectin (α5β1) and the multi-specific laminin, collagens and fibronectin (α3β1) receptors[13]. Nevertheless, the function of individual ECM components, their involvement in cell adhesion as well as the molecular processes mediating growth and invasion of malignant gliomas are not properly characterized.

Several experimental evidences suggest that cell surface proteoglycans are also involved in tumor growth and progression. Highly sulfated glycosaminoglycans (GAGs) and the sulfated polysaccharide fucoidan, stimulate melanoma cell invasion *in vitro*[14]. On the other hand, inhibition of GAGs' sulfation promoted by sodium chlorate treatment reduced the proliferation and adhesion of the leukemic cell line WeHi-3B[15] and C6 glioma cells[16]. Moreover, heparan sulfate proteoglycans have been suggested to play a role in cell adhesion as positive modulators of cell proliferation in Chinese hamster ovary cells[17].

In the present study we have evaluated the role of ECM components in the adhesion, of glioma cells *in vitro*. We report that the addition of the GAGs, heparin and chondroitin sulfate affected C6 cells adhesion to laminin and fibronectin in a specific way in contrast to type IV collagen. In contrast, C6 glioma detachment from these ECM proteins was not altered by GAGs. We also demonstrate an involvement of heparin and chondroitin sulfate in regulating glioma proliferation and migration on ECM proteins. The results suggest a role of GAGs and proteoglycans in regulating glioma adhesion to ECM components as well as proliferation and migration according to the ECM composition.

## Results

**Figure 1:**
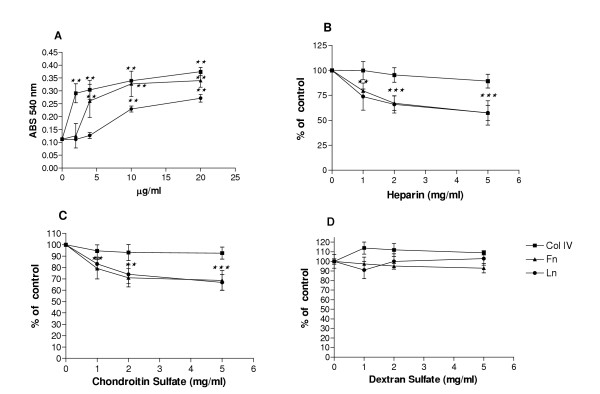
Glycosaminoglycans (GAGs) Effects on C6 Glioma Adhesion to extracellular matrix (ECM) proteins. (A) Dose dependency of C6 glioma adhesion to ECM-coated wells. Wells were coated with different concentrations of type IV collagen (Col IV), Laminin (Ln) or Fibronectin (Fn). Adhesion assays were performed as described in Methods. Results, expressed in Absorbance at 540 nm, were compared with the adhesion in the absence of ECM. Each point represents the mean of four independent experiments performed in triplicate ± SD. Effects of (B) Heparin, (C) chondroitin sulfate and (D) dextran sulfate on C6 Cells adhesion to ECM proteins. Results were compared with cells seeded on ECM substrate only (controls: 100%). Stars in B and C are equivalent for adhesion on laminin or fibronectin. Each point represents the mean of four independent experiments performed in triplicate ± SD. **p < 0.001, ***p < 0.0001 when compared to controls.

The three ECM proteins analyzed, type IV collagen, laminin and fibronectin, induced C6 cell adhesion in a dose-dependent manner (Figure 1A). At the lower ECM concentration (2 μg/ml), C6 cells presented greater adhesion to type IV collagen that was significantly higher (160%) than control (absence of ECM). In contrast, 2 μg/ml of fibronectin or laminin did not display considerable effect on cell adhesion. However, at the concentration of 4 μg/ml, both type IV collagen and fibronectin significantly stimulated C6 cell adhesion (+171% and +133%, respectively) whereas laminin did not affect it. All three ECM proteins presented the maximum adhesion effect at 10 μg/ml, after which a plateau was reached. At this concentration, type IV collagen improved C6 cell adhesion in 203%, fibronectin in 193%, and laminin in 105%. Laminin was the less adhesive substrate to C6 cells in all concentrations analyzed. Similar adherence activity of C6 cells were observed in the absence of ECM molecule and with BSA only (data not shown).

In order to analyze if GAGs could control C6 attachment to ECM molecules, we performed adhesion assays in the presence of heparin or chondroitin sulfate (Figure 1B, C). The addition of heparin significantly impaired cellular attachment to both laminin and fibronectin in a dose-dependent manner (Figure 1B). The higher heparin concentration (5 mg/ml) promoted 42% of inhibition in C6 cell adhesion to laminin and 42% to fibronectin. No inhibitory effect was observed in the attachment to type IV collagen. We also analyzed the attachment of C6 cells to ECM proteins in presence of commercial chondroitin sulfate to verify if other GAG could have similar effects as observed for heparin (Figure 1C). Interestingly, the addition of chondroitin sulfate inhibited C6 adhesion to laminin and fibronectin in a dose dependent manner, similarly to heparin. The higher chondroitin sulfate concentration (5 mg/ml) produced inhibition of 33.2% in C6 cell adhesion to laminin and 31.4% to fibronectin. Chondroitin sulfate did not affect C6 cell attachment to type IV collagen. In addition, both heparin and chondroitin sulfate did not significantly alter C6 adhesion in the absence of ECM molecules, to culture dish plastic (100% ± 10 and 105% ± 8 of adhesion at 5 mg/ml, respectively) or BSA-coated well (92% ± 7 and 95% ± 9 of adhesion at 5 mg/ml, respectively). After that, we investigated the influence of dextran sulfate on C6 adhesion, as a control polyanion (Figure 1D). Dextran sulfate did not alter C6 cells attachment to any ECM protein analyzed. C6 cells adhesion to the culture dish plastic or BSA-coated well were also not affected by dextran sulfate (95% ± 10 and 99% ± 11 at 5 mg/ml, respectively). The results could suggest that heparin and chondroitin sulfate are involved in C6 glioma adhesion to fibronectin and laminin in a specific way. Nevertheless, these GAGs seems do not affect glioma adhesion to collagen IV. These findings could be explained by the presence of proteoglycan binding sites in fibronectin and laminin molecules but not to collagen[18].

To further verify the involvement of GAGs in the modulation of glioma interaction with substrate, we analyzed cellular detachment (Table 1). It is interesting to note that the addition of GAGs and dextran sulfate (alone or in combination) to C6 cells attached to ECM molecules did not induce cell detachment. GAGs and dextran sulfate also have not effect on glioma detachment from culture dish plastic or BSA coated wells. Taken together, the results could suggest that heparin and chondroitin sulfate specifically regulate the initial phase of C6 cells adhesion to the substrate composed of laminin and fibronectin, perhaps in the organization or stabilization of focal contacts. However, these GAGs seem do not display significant effect after the complete cellular attachment.

**Table 1:** GAGs effects on C6 Glioma detachment

Substrate	GAG	% Detachment
Type IV Collagen	Heparin	0% ± 0.14
	Chondroitin Sulfate	7.5 % ± 0.7
	Dextran Sulfate	0% ± 0.2
Fibronectin	Heparin	5% ± 0.9
	Chondroitin Sulfate	4% ± 0.6
	Dextran Sulfate	0% ± 0.2
Laminin	Heparin	0% ± 0.9
	Chondroitin Sulfate	0% ± 0.12
	Dextran Sulfate	0% ± 0.5
Culture dish plastic	Heparin	7% ± 0,2
	Chondroitin Sulfate	5% ± 0,1
	Dextran Sulfate	2% ± 0,3
BSA	Heparin	0% ± 0.1
	Chondroitin Sulfate	5% ± 0,7
	Dextran Sulfate	4% ± 0,4

Wells were coated with 10 μg/ml of type IV collagen, Fibronectin or Laminin. Detachment assays were performed as described. Results were compared with cells seeded on ECM substrate only. Coating with BSA or the culture dish plate was used as controls. Each point represents the mean of three independent experiments performed in triplicate ± SD.

**Figure 2:**
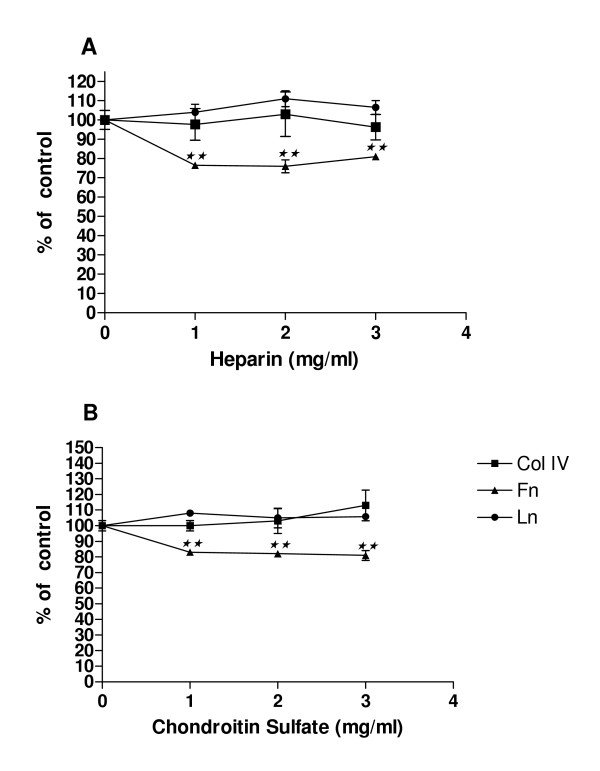
Effects of (A) Heparin and (B) chondroitin sulfate on C6 Cells proliferation. Wells were coated with 10 μg/ml of type IV collagen (Col IV), Laminin (Ln) or Fibronectin (Fn). Proliferation assay was performed by MTT test as described in Methods. Each point represents the mean of three independent experiments performed in triplicate ± SD. **p < 0.001 when compared to cells seeded on ECM substrate only (controls: 100%).

We hypothesized if heparin and chondroitin sulfate treatment of C6 glioma cells adhesion could somewhat be related to modifications in cell proliferation. To test this we analyzed C6 cells proliferation when cultured on ECM proteins in the presence of these GAGs (Figure 2). We observed that heparin significantly reduced C6 cell proliferation on fibronectin as substrate at about 24% C6 in all concentrations analyzed (Figure 2A). In contrast, this GAG did not alter glioma proliferation when cultured on type IV collagen or laminin coated wells. The effects of chondroitin sulfate treatment on C6 cells proliferation were similar to that of heparin (Figure 2B). Chondroitin sulfate significantly impaired about 20% C6 cell proliferation on fibronectin in a similar proportion at the three concentrations tested. On the other hand, cell proliferation on laminin and type IV collagen was not altered. In addition, both heparin and chondroitin sulfate did not affect C6 cell proliferation in the absence of ECM molecules (data not shown).

**Figure 3:**
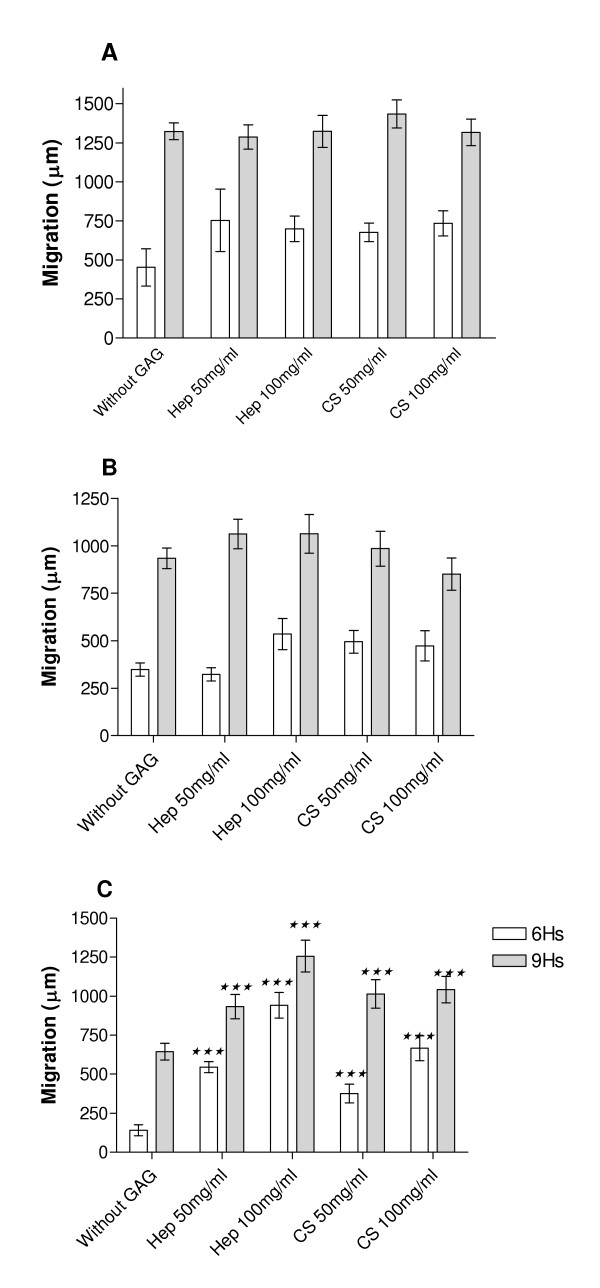
Migration of C6 cells on (A) Type IV Collagen, (B) Fibronectin or (C) Laminin. Wells were coated with 10 μg/ml of each ECM molecule. Cell migration was analyzed at 0, 6 or 9 hour. Quantitative migration measurements were calculated as the increase in the ratio beyond the initial radius of the cell population. CS (chondroitin sulfate), Hep (heparin). Each point represents the mean of three independent experiments performed in triplicate ± SD. ***p < 0.0001 when compared to cells seeded on ECM substrate only (without GAG).

Afterward, we investigated if GAGs also affect C6 glioma migration on ECM (Figure 3). To eliminate possible influence of proliferation, we established as nine hours as time limit in migration assay. The C6 glioma cells migration on different substrates increased over time. Amongst the ECM proteins, type IV collagen was the most effective in inducing C6 cells migration, followed by fibronectin, and subsequently by laminin. The addition of heparin or chondroitin sulfate at tested concentrations (50 or 100 mg/ml) did not alter cell migration on both type IV collagen (Figure 3A) and fibronectin (Figure 3B). In contrast, these GAGs significantly increased cell migration on laminin substrate different proportions according to the dose (Figure 3C). The most prominent effect was observed at time point of six hour when heparin increased cell migration in 222% and 567% 50 and 100 mg/ml, respectively, when compared to the control (without GAG). At this time point, chondroitin sulfate also presented expressive effect in stimulating cell migration (+166% and +373% at 50 and 100 mg/ml, respectively). C6 cell migration without ECM proteins was not affected by both GAGs (data not shown).

## Discussion

Tumor-stroma interactions play a pivotal role in regulation tumor progression and malignance[19]. We had shown previously that undersulfation of GAGs reduces the proliferation of the leukemic cell line Wehi 3B[15] and C6 glioma cells[16] suggesting a regulatory role of proteoglycans on cellular processes involved in tumor progression. We report here that GAGs are also involved in the control of glioma cell adhesion, proliferation and migration on ECM proteins.

It has long been appreciated that some of the earliest steps of neoplastic transformation involve changes in tissue interaction, including alterations in cell adhesion. Loss of cell adhesion was postulated as involved in genomic instability and related to the invasive behavior and motility of malignant cells[4]. Among ECM proteins, laminin was shown as the most potent stimulator of glioma migration and invasion[10,20]. In particular, laminin mediate C6 glioma cells invasion both *in vitro *and *in vivo *[10]. Mahesparan and co-workers[20] reported that laminin was the most efficient ECM protein in inducing cell migration of three different human glioma cell lines (U-373 MG, A-172 MG and HF-66), followed by fibronectin, while type IV collagen did not promote cell migration. In our experiments, however, we observed an inverse correlation with rat C6 glioma regarding both adhesion and migration: type IV collagen was the most potent substrate, followed by fibronectin and somewhat weaker attachment and migration on laminin. It is possible that the different adhesion ability of C6 cells to ECM proteins may be related to its migratory capacity.

For cell adhesion and migration, specific cell surface receptors that interact with ECM components are needed. Cell-surface heparan sulfate[21] or chondroitin sulfate[18] proteoglycans have been implicated in cell attachment via interactions with ECM proteins, such as laminin and fibronectin, and integrins. The syndecan family of transmembrane proteoglycans can act as co-receptors to modulate integrin-mediated cell-matrix adhesion. Together with integrins, they form a dual receptor system active in cell-matrix adhesion[22]. Cell attachment and spreading can be promoted through integrin interactions with the integrin-binding domain of fibronectin. Additional binding of a heparin-binding domain of fibronectin to a cell surface heparan sulfate proteoglycans (HSPG) promotes focal adhesion formation by activating PKC[23]. The HSPG involved appears to be exclusively syndecan-4[23] that binds to fibronectin via its GAG side chains[24].

Several studies have suggested that cell surface proteoglycans are involved in the control of glioma cell growth, adhesion and invasion. The chondroitin sulfate lectican *BEHAB *is up-regulated in malignant gliomas and derived cell lines, and was proposed to play a role in brain tumor invasion[19]. C6 glioma cells transfected with appican display dramatic change in their phenotypic appearance as well as increased cell adhesion [25]. In addition, we have previously demonstrated that sodium chlorate treatment, an inhibitor of GAG's sulfation, reduces C6 cells proliferation and adhesion to ECM proteins further suggesting that cell surface highly sulfated components act as co-receptor for glioma cell attachment to ECM[16]. In the present paper, we observed that the addition of heparin or chondroitin sulfate to the culture dish together within cells impaired C6 adhesion affecting the earliest steps of C6 cell adhesion, possibly due to the competition with GAG chains of cell surface proteoglycans to fibronectin and laminin binding sites. We could suggest that the specific interactions of GAGs with fibronectin and laminin affect the organization of focal adhesions necessary to promote cell adhesion. In contrast, addition of heparin or chondroitin sulfate after cells completely attached to substrate did not promote cell detachment possibly because focal adhesion points were already established and the addition of GAGs were not sufficient to disturb them. In fact, it has been reported that ECM ligands bind to heparan sulfate proteoglycans and produce actin filament reorganization even in the absence of integrin occupancy[26]. The interactions of heparan sulfate chains with extracellular ligands may help the formation of proteoglycan clusters, which appears to be critical for focal adhesion formation[26].

Moreover, it was reported that cell growth is anchorage dependent[27]. Thus, the reduction in C6 cell proliferation on fibronectin promoted by heparin and chondroitin sulfate observed in our experiments could be consequence of the altered cell adhesion. In addition, highly sulfated GAGs, heparin and heparan sulfate, and sulfated polysaccharide, fucoidan, have been reported stimulating tumor cell invasion *in vitro*, due to a stimulation of the proteolytic cascade of plasminogen activation[14]. This suggests that sulfated GAGs liberated by tumor cells, mediate ECM degradation amplifying pericellular plasminogen activation and locally enhancing tumor cell invasion in a positive feedback. In our experiments we observed that both chondroitin sulfate and heparin induced C6 cell migration on laminin substrate in different proportions according to the dose, with the most prominent effect at the fist 6 hours. These results are consistent with the influence of the GAGs on the earliest steps of cell adhesion to ECM proteins (Figure 1 and Table 1). It is possible that GAGs induce the organization of adhesion contacts in C6 cells that specifically lead to proliferation or migration when maintained on fibronectin or laminin, respectively.

## Conclusion

One of the fundamental properties of pericellular proteoglycans is to interact with other molecules, thereby modulating cell adhesion, proliferation, and differentiation or the structural characteristics of tissues[22]. Indeed, we suggest that cell surface proteoglycans may function as coordinators of glioma cell interactions to microenvironment possible acting in the organization focal adhesions. Specific interactions of cell surface proteoglycans with ECM proteins may differently control glioma cell adhesion leading to alterations in proliferation or migration.

## Methods

### C6 glioma cell culture

The rat glioblastoma C6 cell line were a gift from Dr. Vivaldo Moura Neto (Universidade Federal do Rio de Janeiro, Rio de Janeiro, Brazil) and cultured as previously described[16]. Cells were grown in Dulbecco's modified Eagle's medium (DMEM, Sigma, St. Louis) supplemented with 5% fetal calf serum (Fazenda Pigue, Brazil), 100 mg/ml streptomycin and 100 U/ml penicillin (all from Sigma) in a humidified incubator at 37°C in a 5% CO_2_, 95% air atmosphere (standard conditions). Cells were harvested with 0.125% trypsin/0,78 mM EDTA, when they reached confluence.

### ECM coating and adhesion assay

Culture plates (Corning, NY, USA; 96 well) were coated with mouse type IV collagen, laminin or fibronectin (Gibco BRL, Grand Island, NY; 2 to 20 μg/ml) dissolved in phosphate buffered saline (PBS) pH 7.4 (12 hours, 4°C). Wells were washed with PBS, blocked with 2% BSA in PBS (1 hour, 37°C). Cellular adhesion was performed as previously described[16]. Briefly, cells were detached by trypsinization, washed in PBS, suspended in DMEM and seeded into ECM-coated 96-well plates (10^5 ^cells/well). After 2 hours of incubation under standard culture conditions, the non-adherent cells were removed by PBS washing. Attached cells were fixed (4% paraformaldehyde) and stained with 0.5% crystal violet dissolved in 20% methanol. The plates were then washed (PBS) and stain was eluted from cells with 0.1 M sodium citrate, pH 4.2 in 50% ethanol. The amount of stain was analyzed by optical density (OD) in ELISA wells microplate reader (Model ELX800, Bio-Tek Instruments) at 540 nm. In some experiments heparin, chondroitin sulfate or dextran sulfate (all from Sigma, 1 to 5 mg/ml) were added to the attachment medium at the same time as C6 cells were inoculated to the plates previously coated with 10 μg/ml of type IV collagen, laminin or fibronectin. For the detachment assays, cells were prepared as for the adhesion experiment, seeded on ECM-coated wells (5 × 10^4 ^cells/well) and incubated for 3 h in standard culture condition when most of them were attached. After PBS washing, 5 mg/ml of heparin, chondroitin sulfate or dextran sulfate were added and the cultures were incubated for additional 2 hours in standard conditions. Wells were them washed, and the attached cells fixed, stained and processed as described for adhesion assays. Cell detachment was calculated by the difference from the percentage of adherent cells in relation to controls (ECM-coated well without GAG).

### MTT assay

This method has been extensively used to determine glial cell proliferation[16,28,29]. C6 cells were plated (10^4 ^cell/well) in 96-well culture plates previously coated with 10 μg/ml ECM proteins as described above in the presence of heparin or chondroitin sulfate (1 to 3 mg/ml) and cultured for 2 days. Viable cells were quantified by MTT colorimetric assay for mitochondrial dehydrogenase[30].

### Migration assay

A monolayer migration assay was used to quantify locomotion of C6 cells on ECM. Cells (1.5 × 10^5^) were seeded into 13 mm coverslips. After overnight incubation in standard culture conditions, coverslips containing confluent monolayers were transferred to the center of 35 mm wells (with cells up) previously coated with 10 μg/ml of ECM proteins as described above. Fresh DMEM containing heparin or chondroitin sulfate (1 to 3 mg/ml) was added. Cells were maintained in standard culture conditions except during brief microscopic examinations. Migration was calculated by serial measurements of the ratio beyond the initial cell population (cells that migrate out of the coverslip to ECM substrate). The analysis were performed at 0, 6, and 9 hours, using a micrometric ocular (Olympus) attached to an Olympus CK 40 microscope.

### Statistical analysis

The significance of differences was evaluated by means of One-way ANOVA with Newman-Keuls or Dunnett as post-tests using the GraphPad InStat program (San Diego California USA).

## List of abbreviations used

**BSA **– Bovine serum albumin

**DMEM **– Dulbecco's modified Eagle's medium

**ECM **– Extracellular matrix

**GAG **– Glycosaminoglycans

**GBM **– Glioblastoma multiforme

**HSPG **– heparin sulfate proteoglycans

**PBS **– Phosphate buffered saline

## Authors' contributions

CBNMA carried out cell culture, detachment, adhesion, proliferation and migration assays. BLS carried out cell culture and adhesion assays. AGT and MAS conceived the study and participate in the design and coordination. All authors participate in the analysis of data and discussion of the results. All authors read and approved the current version of the manuscript.
